# Terpene-enhanced olaminogel for superior vaginal permeation: robust assessment through in vitro, microbiological, ex vivo, and in vivo evaluations

**DOI:** 10.1007/s00210-025-04838-w

**Published:** 2025-12-19

**Authors:** Sadek Ahmed, Dina Mehana, Heba Attia, Manal M. El-Ashmoony

**Affiliations:** 1 Department of Pharmaceutics and Industrial Pharmacy, Faculty of Pharmacy, Cairo University, Cairo, Egypt; 2 Ain Shams General Hospital, Cairo, Egypt; 3 Department of Microbiology and Immunology, Faculty of Pharmacy, Cairo University, Cairo, Egypt

**Keywords:** Terconazole, Olaminogel, Pseudo-plastic, Time to kill assay, Kinetic analysis, In vivo permeation

## Abstract

Vulvovaginal fungal infections remain a major therapeutic challenge due to poor drug penetration, recurrence, and limited efficacy of conventional formulations. Harnessing nanotechnology with a novel delivery platform offers a promising strategy to overcome these barriers. In this study, an innovative olaminogel was designed as an advanced nanocarrier for terconazole (TCZ) to enhance local antifungal therapy via the vaginal route. The formulation was prepared using the ethanol injection method and systematically optimized through a 2^3^ factorial design considering limonene-to-surfactant ratio (factor A), oleylamine-to-drug ratio (factor B), and oleic acid-to-surfactant ratio (factor C). Optimization targeted maximal entrapment efficiency (EE%), minimal particle size (PS), and stable zeta potential (ZP). The optimized olaminogel achieved an EE% of 82.11, nano-metric PS of 217.25 nm, and a ZP of − 33.05 mV. TEM confirmed well-formed vesicles, while FTIR verified successful encapsulation. Further in vitro characterization revealed pseudo-plastic rheology, sustained biphasic drug release, and stability for 3 months. Mucoadhesion testing demonstrated strong adhesion of the olaminogel to vaginal mucosa. Ex vivo permeation across rabbit vaginal mucosa demonstrated significantly deeper penetration (180 µm vs. 55 µm) compared with plain TCZ gel, corroborated by in vivo CLSM imaging. Histopathological studies further confirmed biocompatibility and absence of irritation. Importantly, microbiological evaluation revealed markedly reduced MIC and MFC values, alongside an accelerated fungicidal effect, outperforming TCZ control. Collectively, these findings highlight olaminogel as a novel and potent intravaginal nanocarrier capable of improving drug retention, mucosal penetration, and antifungal efficacy, thereby presenting a next-generation platform for safe and effective management of vaginal fungal infections.

**Graphical Abstract:**

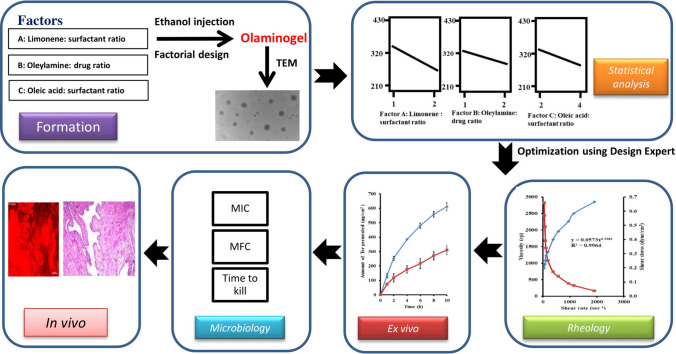

## Introduction

Vaginal infections, particularly fungal vaginitis, are among the most prevalent gynecological disorders affecting women globally. These infections are often caused by *Candida albicans*, a pathogenic yeast responsible for both superficial and invasive candidiasis, which can manifest as acute or chronic and significantly impair quality of life, especially in immunocompromised individuals. Vaginal candidiasis continues to pose a major public health challenge, especially in low-resource settings, where restricted access to modern treatments and growing resistance of *Candida* strains to standard antifungals hinder effective management (Ahmed et al. 2024b). Traditional antifungal therapies, including terconazole (TCZ), an imidazole antifungal agent, often suffer from limitations such as poor water solubility, low mucosal permeability, and undesirable local irritation. Furthermore, overuse and improper administration of antifungal drugs can contribute to the emergence of drug-resistant *Candida* strains, reducing treatment effectiveness and increasing recurrence rates. These challenges emphasize the need for advanced drug delivery systems capable of improving drug solubility, stability, and localized therapeutic action while minimizing side effects (Ahmed et al. 2025b).

The rapid progress in nanotechnology has opened new horizons for drug delivery, offering innovative approaches to address the drawbacks of conventional dosage forms (Elmahboub et al. 2025; Farag et al. 2025). Advanced pharmaceutical delivery platforms are engineered to improve therapeutic outcomes and mitigate unwanted side effects by precisely regulating the spatiotemporal release of active pharmaceutical ingredients (Ahmadi et al. 2023). These sophisticated techniques employ various biomaterials, including polymers, lipids, and nanoparticles, to encapsulate medications, thereby safeguarding them from premature degradation and enabling targeted transport to specific biological sites (M. Saeedi et al. 2024). By surmounting the inherent drawbacks of traditional administration routes, such as low bioavailability or a brief biological half-life, these systems enable a more accurate and customized approach to pharmacotherapy (Omidi et al. 2025). Consequently, the continuous innovation in drug delivery methodologies has the potential to enhance patient adherence to treatment regimens, reduce dosing frequency, and fully exploit the therapeutic promise of both established and emergent medicinal agents (Majid Saeedi et al. 2022).

In this context, nano-vesicular systems have gained attention for their ability to encapsulate hydrophobic drugs such as TCZ, enhancing their permeability and prolonging retention at the site of infection. To address the limitations of traditional systems, surface modification techniques, particularly the incorporation of capping agents, have emerged as a strategic approach to regulate nanoparticle characteristics and ensure stability under physiological conditions (Hassan et al. 2023; Osanloo et al. 2018). Capping agents such as oleylamine are amphiphilic molecules with a high affinity for particle surfaces, functioning to prevent overgrowth, reduce aggregation, and maintain a defined size distribution. Their steric hindrance effect stems from covalent or ionic interactions with other excipients, improving the overall uniformity and biocompatibility of the formulation (Ahmed et al. 2022). Oleylamine, a long-chain primary amine, is known to interact with fatty acids like oleic acid, forming stable carboxylate complexes that support vesicle integrity and prevent coalescence during storage or application (Chen et al. 2007; Shaimaa Mosallam et al. 2022). Limonene, a naturally occurring monoterpene, has been widely recognized for its capacity to act as an effective absorption enhancer across mucosal barriers. Its lipophilic character enables close interaction with biological membranes, where it disrupts the ordered arrangement of lipid bilayers, leading to increased membrane fluidity and reduced lipid packing (Ahmed et al. 2024a). Such structural modification facilitates easier translocation of encapsulated drugs across the mucosal surface. In addition, limonene is capable of forming hydrogen bonds with membrane components, further destabilizing bilayer integrity and enhancing permeability (Albash et al. 2022). Through these combined mechanisms, limonene significantly improves drug diffusivity, prolongs mucosal penetration, and ultimately enhances local bioavailability and therapeutic performance (Nemr et al. 2024). Complementing this action, Span 60, a non-ionic surfactant, contributes to vesicle deformability and enhances bilayer cohesion, supporting consistent drug encapsulation and release. This study introduces olaminogel as a novel nanocarrier system for intravaginal delivery of terconazole, addressing the persistent challenges of poor mucosal penetration and limited efficacy of conventional formulations. By combining nanostructured vesicles with limonene as a natural permeation enhancer and oleylamine as a capping stabilizer, the system achieves strong mucoadhesion, sustained release, and deeper tissue penetration. Such an innovative approach offers a next-generation therapeutic platform for more effective management of vulvovaginal fungal infections.

In line with this objective, the current research focused on the development and optimization of terconazole (TCZ)-loaded olaminogel using the ethanol injection method. The formulation strategy incorporated oleylamine as a capping agent, limonene to enhance mucosal permeability, and Span 60 as a surfactant to stabilize the vesicular system. A 2^3^ factorial design was adopted to systematically evaluate the effects of critical formulation parameters on key attributes including entrapment efficiency (EE%), particle size (PS), polydispersity index (PDI), and zeta potential (ZP). The optimized nanocarrier was extensively characterized using techniques such as Fourier-transform infrared spectroscopy (FTIR) and transmission electron microscopy (TEM). Stability over short-term storage and in vitro release behavior were also assessed. Furthermore, the formulation’s therapeutic potential was explored via microbiological assays, ex vivo permeation analysis, in vivo vaginal uptake using confocal laser scanning microscopy (CLSM), and histological evaluation. Collectively, the findings underscore the potential of olaminogel as a site-specific, safe, and efficient nanocarrier for intravaginal TCZ delivery.

## Materials and methods

### Materials

Terconazole (TCZ) was generously provided by SEDICO Pharmaceutical Company (Cairo, Egypt). Essential formulation components including oleylamine, limonene, Span 60, Rhodamine B, and dialysis membranes (with a molecular weight cut-off of approximately 14,000 Da) were obtained from Sigma Chemical Company. Oleic acid and 95% ethanol, both of analytical reagent grade, were purchased from El Nasr Pharmaceutical Chemicals Co. (Cairo, Egypt). All other reagents and solvents used throughout the study were of analytical grade and employed as received.

### Experimental animals

Adult female albino rabbits were selected as the in vivo model for this investigation. The animals were individually housed in a controlled laboratory environment maintained at 25 ± 2 °C, with regulated humidity and a 12-h light/dark cycle. They were granted unrestricted access to standard dry feed and clean drinking water. Prior to the start of the experiments, all rabbits underwent clinical evaluation to ensure they were in good health and free from any vaginal infections. The experimental procedures received ethical approval from the Research Ethics Committee at the Faculty of Pharmacy, Cairo University, Egypt (approval no. PI 3737). All animal handling and procedures conformed to the guidelines outlined in the U.S. National Institutes of Health’s Guide for the Care and Use of Laboratory Animals (NIH Publication No. 85–23, revised 2011), and followed the ARRIVE guidelines for responsible reporting of animal-based research (Abdurrahman M. Fahmy et al. 2025).

### Method

#### Experimental design

A 2^3^ factorial design was employed using Design-Expert® software (Stat-Ease Inc., Minneapolis, MN, USA) to systematically investigate the effects of selected formulation variables on critical physicochemical characteristics of the developed system (Rosini and Pollegioni 2020). The independent variables examined were (A) limonene-to-surfactant ratio, (B) oleylamine-to-drug ratio, and (C) oleic acid-to-surfactant ratio. The measured outcomes (dependent variables) included Y1: entrapment efficiency (EE%), Y2: particle size (PS, nm), Y3: polydispersity index (PDI), and Y4: absolute zeta potential (ZP, mV). The formulation was optimized by targeting the maximum in EE% and ZP, alongside the minimum in PS and PDI, to achieve the most efficient combination of variables. The design matrix, as outlined in Table 1, summarizes the levels of the independent variables, the experimental results for each response, and the desirability criteria utilized in the optimization approach (Farag et al. 2025; Patel et al. 2009).

**Table 1:** Factorial levels of studied independent variables together with measured responses and their desirability constraints

Factor (independent variable)	Level	
	** − 1**	** + 1**
A: Limonene: surfactant ratio B: Oleylamine: drug ratio C: Oleic acid: surfactant ratio	1:1 1:1 2:1	2:1 2:1 4:1
**Response (dependent variable)**	**Desirability constraints**	
Y1: EE% Y2: PS (nm) Y3: PDI Y4: ZP (absolute value) (mV)	Maximize Minimize Minimize Maximize	

*EE%*, percent entrapment efficiency; *PDI*, poly-dispersity index; *PS*, particle size; *ZP*, zeta potential

#### Preparation of terconazole-loaded olaminosomes

Olaminosomes encapsulating terconazole (TCZ) were prepared using the ethanol injection technique, with modifications based on earlier protocols (Nasr and Sammour 2020). Briefly, 10 mg of TCZ and 25 mg of Span 60 were combined with specific proportions of oleylamine, limonene, and oleic acid and then dissolved in 10 mL of ethanol to form the organic phase. This solution was heated to 60 °C using a water bath to ensure thorough solubilization. Simultaneously, the aqueous phase, consisting of double-distilled water in a volume twice that of the organic phase, was also pre-heated to 60 °C (Bender et al. 2024). The ethanoic mixture was then gradually added to the aqueous phase under continuous magnetic stirring (MSH-20D, GmbH, Berlin, Germany). Stirring was maintained until the complete removal of ethanol, leading to the self-assembly of olaminosomal vesicles. The resulting dispersions were stored at 4 °C pending further evaluation (Ahmed et al. 2025c; Al-Mahallawi et al. 2014). The formulation details of the prepared formulae (F1–F8) are presented in Table 2.

**Table 2:** Composition of Ter-loaded olaminosomes with their measured responses (*n* = 3 ± SD)

Formula	Factors						
	**A: Limonene: surfactant ratio**	**B: Oleylamine: drug ratio**	**C: Oleic acid: surfactant ratio**	**Y1: EE%** **(mean ± SD)**	**Y2: PS (nm)** **(mean ± SD)**	**Y3: PDI** **(mean ± SD)**	**Y4: ZP (mV)** **(mean ± SD)**
**F1**	1:1	1:1	4:1	75.41 ± 1.82	331.0 ± 15.84	0.39 ± 0.02	− 26.80 ± 0.14
**F2**	2:1	2:1	2:1	69.38 ± 8.52	287.8 ± 8.56	0.31 ± 0.04	− 24.85 ± 0.78
**F3**	1:1	2:1	2:1	72.11 ± 1.69	340.5 ± 22.34	0.36 ± 0.02	− 25.00 ± 0.14
**F4**	1:1	1:1	2:1	66.83 ± 3.29	426.9 ± 21.57	0.40 ± 0.01	− 20.50 ± 0.99
**F5**	2:1	2:1	4:1	82.11 ± 2.19	217.3 ± 6.58	0.32 ± 0.10	− 33.05 ± 0.92
**F6**	2:1	1:1	2:1	68.84 ± 2.91	293.5 ± 3.96	0.39 ± 0.06	− 24.50 ± 0.14
**F7**	2:1	1:1	4:1	78.49 ± 1.45	271.5 ± 23.69	0.30 ± 0.01	− 27.90 ± 0.85
**F8**	1:1	2:1	4:1	84.31 ± 0.96	293.9 ± 30.12	0.44 ± 0.05	− 32.95 ± 0.49

*Ter*, terconazole; *EE%*, percent entrapment efficiency; *PDI*, polydispersity index; *PS*, particle size; *ZP*, zeta potential

#### In vitro characterization of TCZ-loaded olaminosomes

##### Entrapment efficiency (EE%)

The efficiency of terconazole (TCZ) entrapment within the olaminosomal vesicles was assessed by isolating the non-encapsulated drug through ultracentrifugation. Formulations were centrifuged at 21,000 rpm for 1 h at 4 °C using an ultracentrifuge (Sigma 3K30, Sigma Laborzentrifugen GmbH, Germany). The supernatant containing free TCZ was carefully collected and analyzed at 254 nm using a UV–Vis spectrophotometer (Shimadzu UV-1601 PC, Japan) (Ahmed et al. 2025g). Drug concentration was determined based on a validated standard calibration curve (*n* = 3, *R*^2^ = 0.9994). Entrapment efficiency was calculated using the following formula (Elsayed and Sayed 2017; Sayed et al. 2020).

1 $$EE\%=\frac{(Total\;amount\;of\;TCZ-Total\;amount\;of\;freeTCZ)}{Total\;amount\;of\;TCZ}\times100$$

The total TCZ reflects the full dose of drug introduced into the formulation process, whereas the free TCZ denotes the unencapsulated fraction quantified in the supernatant following centrifugation.

##### Particle size (PS), polydispersity index (PDI), and zeta potential (ZP)

The vesicular size, distribution uniformity (PDI), and surface charge (ZP) of the formulations were evaluated at 25 °C using dynamic light scattering (DLS) at a fixed scattering angle of 173° via a Zetasizer Nano ZS (ZEN3600, Malvern Instruments, UK) (Sadek Ahmed et al. 2021; El Hassab et al. 2025). Prior to measurements, each sample was diluted tenfold with distilled water to ensure optimal transparency and dispersion. All readings were conducted in triplicate, and results were reported as mean ± standard deviation (R. Albash et al. 2025).

##### Formulation optimization

Statistical analysis of the experimental data was performed using ANOVA with the aid of Design-Expert® software (Stat-Ease Inc., Minneapolis, MN, USA). The optimization approach relied on a desirability function aiming to maximize both entrapment efficiency (EE%) and zeta potential (ZP), while minimizing particle size (PS) and PDI (Ahmed et al. 2025f). To verify the model’s accuracy, the optimal formulation was re-fabricated under the same experimental conditions, and its characteristics were re-evaluated. The obtained results were compared to predicted values, and the percentage error was calculated to determine the model’s predictive reliability (Sadek Ahmed et al. 2021; Said et al. 2020).

#### Physicochemical characterization of the optimum formula

##### Fourier-transform infrared spectroscopy (FTIR)

FTIR spectroscopy was utilized to investigate potential molecular interactions among formulation components. Fourier-transform infrared (FTIR) spectra were acquired using a Bruker spectrometer (Model 22, Coventry, UK) within the range of 4000 to 500 cm⁻^1^ (Ahmed et al. 2025h). KBr pellet discs were individually prepared for terconazole (TCZ), oleylamine, Span 60, and the final optimized formulation. The resulting spectral profiles were analyzed to identify key functional group vibrations, as well as to detect any peak shifts or changes in intensity that might indicate molecular interactions or structural modifications. Alterations in the spectral features of TCZ or oleylamine within the optimized formulation were interpreted as indicative of drug encapsulation or possible molecular interactions within the olaminosomal matrix (N. F. Younes et al. 2018a).

##### Transmission electron microscopy (TEM)

The surface morphology and structural features of the optimized formula were visualized using transmission electron microscopy (TEM, JEM-2100, JEOL, Japan) operated at an accelerating voltage of 80 kV to capture high-resolution images of the vesicular architecture (Asmaa Ashraf Nemr et al. 2025; N. F. Younes et al. 2018b). A small volume of the diluted formulation was carefully deposited onto a carbon-coated copper grid and left to air dry at room temperature before imaging (Ahmed et al. 2025e).

##### In vitro release and kinetic modeling

The in vitro release behavior of the optimized TCZ-loaded formulation was investigated using the dialysis bag technique. An amount equivalent to 1 mg of terconazole, either from the optimized formula or a drug suspension, was loaded into a dialysis membrane (molecular weight cut-off 12,000–14,000 Da). The membrane was securely sealed and submerged in 50 mL of a release medium composed of phosphate-buffered saline (pH 4.5) containing 10% v/v methanol (Ahmed et al. 2025g). The system was maintained at 37 °C with continuous stirring to simulate physiological conditions. At predetermined time intervals, aliquots were withdrawn for spectrophotometric analysis to quantify TCZ concentration, and an equal volume of fresh medium was added to preserve sink conditions (Weng et al. 2020). The cumulative amount of drug released was plotted against time, and the drug release data were analyzed by fitting them to different kinetic models, such as zero-order, first-order, and Higuchi models, to better understand the mechanism governing the release behavior (Elsayed and Sayed 2017; Nasra et al. 2017).

##### Mucoadhesion study

The mucoadhesive properties of the optimized formula were assessed through its interaction with a mucin dispersion (1% w/v), a standard approach for predicting vaginal retention. Equal volumes of the optimized formula and mucin solution were mixed in a 1:1 ratio and subjected to gentle magnetic stirring for 5 min to ensure uniform contact, after which the mixture was left to equilibrate overnight at room temperature (Asmaa Ashraf Nemr et al. 2024). To investigate the extent of interaction, the zeta potential (ZP) of the mucin solution and the optimized formula were determined individually and then compared with the ZP of the combined system using a Zetasizer (ZEN3600, Malvern Instruments, UK) (Tawfik et al. 2025). A pronounced shift in ZP values following mixing was taken as evidence of electrostatic interactions between the nano-vesicles and mucin chains, thereby confirming the strong mucoadhesive potential of the optimized vaginal formulation (Ahmed et al. 2025g; R. Albash et al. 2021).

##### Stability assessment under short-term storage

The stability of the optimized formula was monitored over a period of 3 months under refrigerated conditions (4–8 °C) (Ahmed et al. 2025e). At the end of the storage period, the formulation was evaluated for any noticeable physical changes and reanalyzed for key parameters including entrapment efficiency (EE%), particle size (PS), and zeta potential (ZP) (Al-Mahallawi et al. 2017; Rofida Albash et al. 2022). These values were statistically compared to those of the freshly prepared formulation using one-way ANOVA. Additionally, to compare the release behavior before and after storage, the similarity factor (ƒ₂) was computed according to the equation below (Abd-Elbary et al. 2008):

2 $$f_2=50.log\{\lbrack1+\left(\frac1n\right)\sum\nolimits_{t=1}^n\left(R_t-T_t\right)^2\rbrack^{-0.5}.100$$

Here, *R*ₜ and *Tₜ* represent the cumulative percentage of TCZ released at time point *t* from the fresh and stored samples, respectively. A calculated ƒ₂ value ranging between 50 and 100 signifies a strong similarity between the two profiles, indicating minimal impact of storage on formulation performance (A. M. Fahmy et al. 2021; Teama et al. 2025).

#### Ex vivo investigations

##### Olaminogel formation

To convert the optimized dispersion into olaminogel, Carbopol 934 was incorporated at a concentration of 1% w/w. The polymer was slowly dispersed under continuous magnetic stirring to achieve uniform distribution and facilitate gel network formation (Sadek Ahmed et al. 2021). Subsequently, triethanolamine (TEA) was carefully introduced dropwise to neutralize the system and adjust the pH, thereby inducing gelation. The prepared mixture was then kept at 4 °C overnight to ensure complete polymer hydration, yielding a clear, homogeneous, and stable gel matrix (Ahmed et al. 2025g).

##### Rheological evaluation

The flow characteristics of the prepared gel were examined using a Brookfield cone and plate viscometer (spindle CPE-41) under controlled temperature conditions (25 ± 1 °C). Approximately 0.5 g of the gel sample was applied to the plate, and measurements were taken across a speed range of 0.5 to 100 rpm, with a 10-s interval between each rotational step. Only torque values within the acceptable range of 10%–100% were considered valid for analysis. The relationship between shear stress, shear rate, and viscosity was plotted to construct a flow curve. To further characterize the gel’s rheological profile, the power law model was applied, described by the equation (A. A. Nemr et al. 2021; Nihal Farid Younes et al. 2024): 

3 $$\tau=K.\gamma^n$$

In this equation, *τ* represents shear stress, *γ* is the shear rate, *K* denotes the consistency coefficient, and *n* indicates the flow behavior index. A value of *n* < 1 reflects pseudoplastic or shear-thinning behavior, typical of formulations that become less viscous with increased shear. A value of *n* = 1 indicates Newtonian flow, while *n* > 1 suggests a dilatant or shear-thickening system (Ahmed et al. 2025a). To provide a more comprehensive understanding of the gel’s non-Newtonian nature, additional rheological models—including Bingham, Casson, and Carreau—were tested. The best-fitting model was determined based on the highest coefficient of determination (*R*^2^), indicating optimal correlation with the experimental data (A. M. Fahmy et al. 2018).

##### Ex vivo permeation study

Permeation experiments were carried out using excised vaginal tissues harvested from healthy adult female albino rabbits (weighing 2–3 kg). Anesthesia was administered via intramuscular injection of ketamine (35 mg/kg) in combination with xylazine (5 mg/kg) [14]. The vaginal tract was surgically removed by carefully separating it from the adjacent perineal structures, ensuring the entire vaginal canal was excised as a single, intact segment. The excised tissue was longitudinally opened to expose the anterior vaginal wall, which was then transversely divided into proximal and distal segments (Satapathy et al. 2023). The proximal section of the excised vaginal tissue was immersed in distilled water and utilized for diffusion studies within 4 h post-excision to preserve tissue viability (Mao et al. 2019). For the ex vivo permeation setup, the tissue was carefully positioned between the donor and receptor chambers of a Franz diffusion apparatus. A sample containing 2 g of the optimized olaminogel formulation (corresponding to 1000 μg TCZ), as well as an equivalent amount of plain TCZ gel, were intravaginally administered using a soft, pliable polyethylene applicator engineered to deliver the preparation both precisely and atraumatically. The selected dose and volume were determined through preliminary trials to provide sufficient mucosal coverage while minimizing the risk of leakage or expulsion, thereby ensuring consistency and reproducibility of the administration protocol (Ahmed et al. 2025g). The samples were placed in the donor compartment. The receptor compartment was filled with methanolic phosphate-buffered saline (10% v/v, pH 4.5), kept at a constant temperature of 37 °C, and agitated at 100 rpm throughout the experiment. At predetermined time points, aliquots were withdrawn from the receptor medium and immediately replaced with fresh buffer to maintain sink conditions. The TCZ content in the collected samples was quantified spectrophotometrically at 254 nm (Ahmed et al. 2025h). Critical permeation metrics, including the cumulative drug amount permeated over 10 h (Q10h), peak steady-state flux (*J*ₘₐₓ), and the enhancement ratio (ER), were calculated using established formulas (Sayed et al. 2018):

4 $$Jmax=\frac{Amount\;of\;drug\;permeated}{Time\times Area}$$

5 $$ER=\frac{Jmax\;(olaminogel)}{Jmax\;(TCZgel)}$$

A plot illustrating the cumulative permeation of terconazole (µg/cm^2^) over time (hours) will be generated to evaluate and compare the permeation profiles of the different tested formulations.

#### Microbiological evaluations

##### Determination of the minimum inhibitory concentration (MIC)

The antifungal activity of both the free drug and its optimized formulation against *Candida albicans* was determined using the broth microdilution technique, in accordance with the Clinical and Laboratory Standards Institute (CLSI) protocol (Clinical and Laboratory Standards 2018). A series of two-fold serial dilutions was prepared in double-strength Sabouraud Dextrose Broth (SDB), yielding 200 µL aliquots of each concentration, ranging from 500 to 0.488 µg/mL. These aliquots were distributed into sterile, U-bottom 96-well microtiter plates for subsequent testing (Ahmed et al. 2025h).

Thirty microliters of a standardized *Candida albicans* (ATCC 60193) inoculum, adjusted to 10^5^–10⁶ CFU/mL, was added to each well. Control groups included a growth control (untreated fungal inoculum), a sterility control (uninoculated broth), and a blank control containing only formulation excipients. Biological and technical triplicates of the experiment were conducted (Ahmed et al. 2025g). Following incubation at 28 ± 2 °C for 48 h, fungal growth was assessed visually and by measuring turbidity at 600 nm using a microplate reader (Biotek, Synergy 2, USA). The MIC was defined as the lowest concentration at which no visible fungal growth was observed.

##### Determination of the minimum fungicidal concentration (MFC)

To evaluate the fungicidal effect, two-fold serial dilutions of both the plain drug and optimized formulation were prepared and mixed with 30 µL of *Candida albicans* inoculum (10^5^–10⁶ CFU/mL) in sterile 96-well plates. After a 48-h incubation at 28 ± 2 °C, 10 µL aliquots from each well were aseptically transferred and spread on Sabouraud Dextrose Agar (SDA) plates. The SDA plates were further incubated for 48 h under the same conditions. The MFC was identified as the lowest concentration at which no visible fungal colonies were observed. All experiments were performed in triplicate, ensuring consistency and reliability of results through both biological and technical replication.

##### Time to kill assay

A modified procedure, adapted from the method described by Osman et al., was utilized to evaluate the fungicidal activity of both the pure drug and its optimized formulation against a reference strain of *Candida albicans* (Osman et al. 2023). Three Eppendorf tubes were filled with 1.5 mL Sabouraud Dextrose Broth (SDB) to serve as the incubation medium. Two tubes were supplemented with the minimum inhibitory concentrations (MICs) of either the pure drug (3.9 µg/mL) or the optimized formulation (125 µg/mL), while the third tube served as the positive control. A volume of 150 µL from a standardized *Candida albicans* suspension (10⁶ CFU/mL) was introduced into each of the three tubes. A baseline sample (0-h timepoint) was collected by serially diluting 10 µL of each mixture with 10 times sterile saline and plating onto Sabouraud Dextrose Agar (SDA). During incubationof the Eppendorf tubes at 28 ± 2 °C, 10 µL aliquots were withdrawn from each tube at 4, 6, 10, 24, 30, 36, 48, and 72 h. Each withdrawn sample was diluted at a 1:10 ratio in sterile saline solution, followed by plating of 10 µL onto SDA plates and incubation under identical conditions. Fungal growth was observed and documented at each predetermined time interval. All assays were performed in three independent replicates to confirm the reliability and reproducibility of the results. The fungicidal time, defined as the shortest time point at which no detectable fungal colonies remained, was recorded to determine the time required for complete fungal eradication.

#### In vivo evaluation of the optimized olaminogel

##### Histological examination

To assess the safety of the optimized olaminogel on vaginal tissues, a histopathological study was conducted using two groups of female albino rabbits. Group I served as the untreated control, while Group II received intravaginally 2 g of the optimized olaminogel (corresponding to 1000 μg TCZ) administered using a soft, pliable polyethylene applicator twice daily for 7 days. General anesthesia was administered via intramuscular injection of ketamine (35 mg/kg) and xylazine (5 mg/kg) (Sayed et al. 2020). At the end of the treatment period, vaginal tissues were carefully harvested, fixed immediately in 10% formalin saline, and embedded in molten paraffin wax. Thin tissue slices were obtained using a rotary microtome and stained with hematoxylin and eosin (H&E). Microscopic analysis was carried out using a digital light microscope (Leica, Cambridge, UK) to detect any structural or cellular alterations indicative of inflammation, irritation, or tissue damage (A. A. Abdelbary et al. 2019).

##### Mucosal penetration via CLSM

The ability of the optimized formulation to penetrate vaginal mucosa was examined using confocal laser scanning microscopy (CLSM). Rhodamine B (RhB), a fluorescent tracer, was incorporated at 0.1% w/w as a model compound in place of terconazole. Female rabbits were divided into two experimental groups (*n* = 3); one received 2 g of a plain RhB gel, and the other was treated with 2 g of the RhB-containing olaminogel using pliable polyethylene applicator. After 6 h of vaginal application, vaginal tissues were dissected, rinsed to remove surface residue, and prepared for microscopic observation. A Zeiss LSM 710 system (Carl Zeiss, Jena, Germany) equipped with argon (485 nm) and helium–neon (595 nm) lasers was used for imaging. Fluorescence intensity and penetration depth were analyzed using LSM software version 4.2 to compare the mucosal distribution of RhB in both treatment groups (Darvin 2023; Kwon et al. 2012).

### Statistical analysis

All experimental outcomes derived from the factorial design and in vivo studies were presented as mean ± standard deviation (SD), based on triplicate measurements. Statistical significance was assessed using one-way analysis of variance (ANOVA), with a *p*-value of less than 0.05 considered statistically significant. When comparing multiple treatment conditions, post hoc analysis using the least significant difference (LSD) method was performed to determine specific differences among groups (Sadek Ahmed et al. 2021).

## Results and discussion

### Analysis of factorial design

A 2^3^ factorial design was employed to systematically explore the effects of three critical formulation parameters on the properties of the developed olaminosomes. The studied independent variables included the ratio of limonene to surfactant (factor A), oleylamine to drug (factor B), and oleic acid to surfactant (factor C). Preliminary trials were conducted to establish suitable upper and lower limits for each factor. The resulting dataset was subjected to statistical analysis using Design-Expert® software. The experimental responses were fitted to relevant mathematical models, which demonstrated strong predictive capability. High coefficients of determination (*R*^2^), along with good agreement between adjusted and predicted *R*^2^ values, affirmed the robustness and reliability of the selected models across all measured outcomes. Additionally, the adequate precision values exceeded the accepted threshold, further validating the robustness of the models (Ahmed et al. 2025c; A. Fahmy et al. 2020; Sayed et al. 2018). Detailed results are presented in Table 3.

**Table 3:** Model analysis for studied responses

Response	*R*^2^	Adjusted *R*^2^	Predicated *R*^2^	Adequate precision	Significant factors
**EE%**	0.9368	0.8894	0.7473	10.126	B, C
**PS (nm)**	0.9230	0.8653	0.6922	11.687	A, B, C
**ZP (mV)**	0.9182	0.8568	0.6727	10.202	B, C

*EE%*, percent entrapment efficiency; *PS*, particle size; *ZP*, zeta potential

### Evaluation of entrapment efficiency (EE%)

**Fig. 1:**
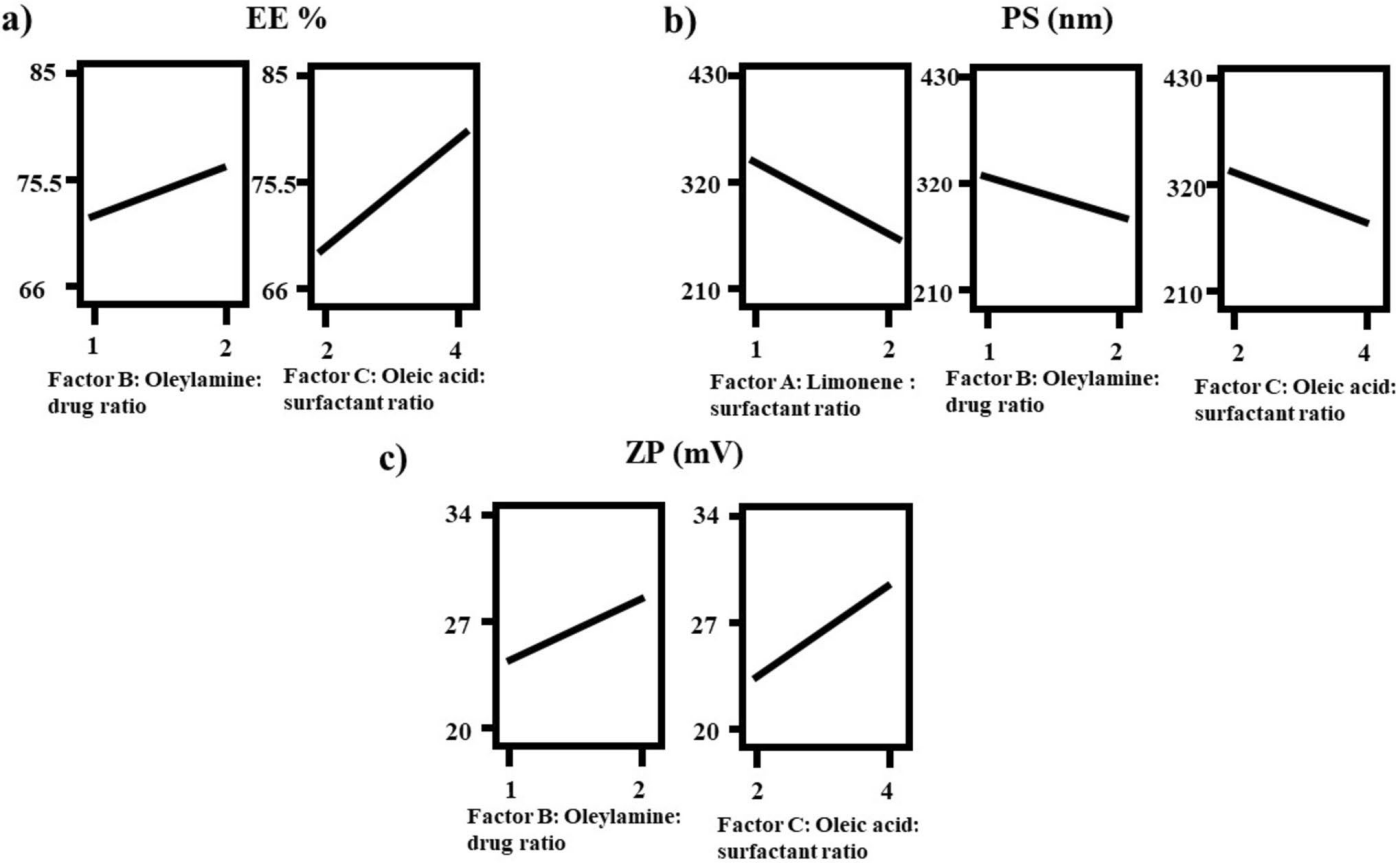
Response-plots for the effect of factor A: limonene: surfactant ratio, factor B: oleylamine: drug ratio, and factor C: oleic acid: surfactant ratio on **a** EE%, **b** PS, and **c** ZP

Maximizing drug entrapment within nanocarriers is essential for improving therapeutic efficacy and sustaining the release of hydrophobic compounds such as terconazole (TCZ). As presented in Table 2, the entrapment efficiency across different formulations varied between 66.83 ± 3.29% and 84.31 ± 0.96%. Analysis of variance (ANOVA) revealed that factor B (the ratio of oleylamine to TCZ) and factor C (the ratio of oleic acid to surfactant) exerted a statistically significant effect on EE% (*p* < 0.05), while factor A did not show a notable influence. These outcomes are visually demonstrated in the interaction plots shown in Fig. 1a and are quantitatively described by the generated regression model.

$$EE\%=74.68+0.019A+2.29B+5.39C.$$

Regarding factor B, the oleylamine: drug ratio significantly impacted entrapment efficiency (EE%). Oleylamine, due to its strong hydrophobicity, effectively solubilizes lipophilic drugs like TCZ and functions as a gelling agent by forming three-dimensional fibrillar networks through non-covalent interactions. These networks assist in trapping large amounts of the surrounding medium, thereby improving the encapsulation efficiency (EE%). Increasing the oleylamine concentration further facilitates the development of these network structures, resulting in enhanced drug retention (Abd-Elsalam and ElKasabgy 2019; Ahmed et al. 2024a, b).

Regarding factor C, the oleic acid: surfactant ratio also demonstrated a considerable influence on EE%. Oleic acid improves TCZ encapsulation by enhancing its solubility and integration within the lipid bilayer. It also interacts with oleylamine to form carboxylate derivatives, contributing to a stronger capping effect and enhanced formulation stability (Subramaniam et al. 2020; Yang et al. 2023). In contrast, factor A (limonene: surfactant ratio) showed no significant effect on EE%. Although limonene offers benefits including the enhancement of solubility, permeability, and nanoparticle stability, its contribution to EE% in this formulation was negligible (Akhavan-Mahdavi et al. 2022). Ahmed et al. reported that the encapsulation efficiency of hydrophobic fenticonazole was markedly improved after incorporation of oleylamine, which in turn enhanced the therapeutic management of ocular candidiasis (Sadek Ahmed et al. 2022). Similarly, Mishra et al. demonstrated that the incorporation of oleic acid significantly improved the entrapment of itraconazole in ultra-deformable transethosomes (Mishra et al. 2022). Overall, these findings highlight the pivotal roles of oleylamine and oleic acid in optimizing drug loading and stabilizing olaminosomal vesicles for effective vaginal delivery of TCZ.

#### Model analysis of particle size (PS)

Smaller particle size (PS) is a pivotal characteristic in vesicular drug delivery systems, facilitating enhanced mucosal penetration and improved therapeutic performance. According to Table 2, the PS ranged from 217.3 ± 6.58 nm to 426.9 ± 21.57 nm, confirming their nanoscale dimension. ANOVA results and Fig. 1b confirmed that all three formulation variables: A (limonene: surfactant ratio), B (oleylamine: drug ratio), and C (oleic acid: surfactant ratio) exerted a statistically significant effect. The significant negative coefficients (*p* < 0.05) associated with the studied variables suggest that higher levels of these factors led to a notable reduction in particle size (PS). This is further supported by the regression equation derived from the experimental design:

$$Particle\;Size(nm)=307.78-40.29A-22.93B-29.38C$$

The negative coefficients for all three factors indicate an inverse relationship with particle size. Specifically, increasing limonene (A) led to a significant reduction in particle size due to its ability to lower interfacial tension and rapidly evaporate during emulsification. Acting as a co-surfactant or part of the oil phase, limonene enhances emulsification efficiency, promoting the formation of smaller and more stable particles. Furthermore, limonene fluidizes the lipid matrix, which facilitates the production of uniformly sized particles. Its plasticizing and solubilizing properties also contribute to better drug dispersion and a decrease in the effective particle size (Ghasemi et al. 2018). Increasing oleylamine (B) contributed to size reduction. As a strong capping agent, oleylamine prevents vesicle agglomeration and enhances uniformity through steric stabilization from its long alkyl chain (Mourdikoudis and Liz-Marzán 2013). Increasing oleic acid (C) also markedly decreased PS by disrupting the lipid bilayer structure, reducing surface tension and rigidity, which promotes the formation of smaller and more flexible vesicles (Ahmed et al. 2024b; Naik et al. 1995). Abd-Elsalam et al. reported that the inclusion of oleylamine significantly reduced particle size, which enhanced the therapeutic efficacy of agomelatine in lowering ocular pressure. This observation is in line with our findings (Abd-Elsalam and ElKasabgy 2019). Furthermore, similar results were reported by Tawfik et al., who demonstrated that increasing terpene concentrations led to the generation of smaller vesicular systems, particularly in zolmitriptan Terpesomes (Tawfik et al. 2023). In another study, Abdulbaqi et al. highlighted the dual role of oleic acid as both a structural component and a permeation enhancer, noting its ability to markedly reduce particle size (Abdulbaqi et al. 2016). These combined influences led to the formation of uniform, nanosized, and stable olaminosomes, which are optimal for efficient and prolonged vaginal delivery of TCZ.

#### Analysis of polydispersity index (PDI)

The polydispersity index (PDI) is a critical parameter used to assess the uniformity of particle size distribution within nanoscale formulations. Lower PDI values, closer to zero, are indicative of a more homogeneous system, which is essential for enhancing formulation stability and ensuring consistency across production batches. According to the data presented in Table 2, PDI values for all formulations ranged from 0.30 ± 0.01 to 0.44 ± 0.05, indicating a relatively narrow size distribution throughout. Statistical analysis using ANOVA showed that none of the formulation variables (A, B, or C) had a significant impact on the PDI (*p* > 0.05), suggesting that particle size uniformity remained largely unaffected by the studied factors. Ahmed et al. reported comparable findings regarding the polydispersity index (PDI), where a low PDI value was indicative of uniform size distribution and confirmed the homogeneity and stability of the developed system (Ahmed et al. 2025e). Collectively, these results confirm that all formulations achieved an adequate level of uniformity, supporting their potential suitability for topical and mucosal drug delivery (Mosallam et al. 2021; Younes et al. 2024).

#### Model analysis of zeta potential (ZP)

Zeta potential (ZP) serves as a key parameter in evaluating the colloidal stability of nanovesicular systems, where values around ± 30 mV or higher typically indicate sufficient surface charge to prevent particle aggregation via electrostatic repulsion. In this study, ZP values, presented in Table 2, ranged from − 20.50 ± 0.99 mV to − 33.05 ± 0.92 mV, reflecting moderate to strong repulsive interactions among vesicles. According to ANOVA results and the graphical representation in Fig. 1c, factors B (oleylamine-to-drug ratio) and C (oleic acid-to-surfactant ratio) significantly influenced ZP (*p* < 0.05), whereas factor A (limonene-to-surfactant ratio) did not exhibit a statistically significant effect. The relationship is described by the regression equation:

$$ZP=26.94+0.63A+2.02B-3.23C.$$

The positive effect of factors B and C indicates that increasing these factors leads to more negative ZP values, which reflects a favorable effect on colloidal stability. Specifically, increasing oleylamine enhances surface charge through amine group adsorption (Özkar and Duman 2017), while oleic acid contributes additional negative charges via its carboxyl group and affects bilayer packing (Verma et al. 2014). These changes improve repulsion between vesicles and reduce aggregation risk. In contrast, limonene had a negligible effect on ZP within the tested concentration range (Marathe et al. 2022). Consistent with our findings, Abd-Elsalam et al. attributed the high negative zeta potential of olaminosomes to the free carboxylic groups of oleic acid (Abd-Elsalam and ElKasabgy 2019). Likewise, Ahmed et al. reported that oleylamine stabilizes capped flexosomes through interfacial adsorption and proton affinity, preventing aggregation and maintaining nanoparticle uniformity (Ahmed et al. 2024b).

### Confirmation of the optimization process

Through numerical optimization, the ideal formulation was determined to include a limonene-to-surfactant ratio of 2:1 (factor A), an oleylamine-to-drug ratio of 2:1 (factor B), and an oleic acid-to-surfactant ratio of 4:1 (factor C), resulting in a high desirability value of 0.956. As shown in Table 4, the actual experimental results were in close agreement with the predicted values, with all percentage deviations falling below 5% (Ahmed et al. 2025e). This high level of accuracy supports the robustness and predictive strength of the applied statistical model. The strong alignment between observed and expected data highlights the reliability of the factorial design and validates the significance of the selected formulation variables. Consequently, the optimized olaminosomal system was deemed suitable for comprehensive evaluation across in vitro,ex vivo, microbiological, and in vivo investigations.

**Table 4:** Characterization of the optimum formula

Response	Y1	Y2	Y4
	EE%	PS (nm)	ZP (mV)
**Observed value**	82.11	217.25	− 33.05
**Predicated value**	82.39	215.19	− 32.82
**% Deviation (absolute)**	0.34	0.96	0.69

*EE%*, percent entrapment efficiency; *PS*, particle size; *ZP*, zeta potential

### In vitro characterization of the optimized formulation

#### Fourier-transform infrared spectroscopy (FTIR)

**Fig. 2:**
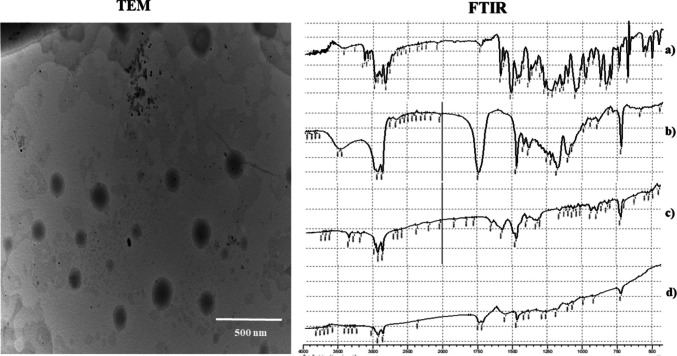
FTIR spectra of pure **a** terconazole, **b** Span 60, **c** oleylamine, and **d** optimum formula. In addition to, TEM of the optimum formula

Figure 2 illustrates the FTIR spectra of pure terconazole (TCZ), oleylamine, and the optimum formula. TCZ exhibited characteristic infrared absorption peaks around 1610.4 cm⁻^1^, 1514.2 cm⁻^1^, 1273.6 cm⁻^1^, and 767.8 cm⁻^1^, which are attributed to imidazole C = N stretching, aromatic C = C vibrations, ether (C–O–C) functional groups, and C–Cl bond stretching, respectively confirming the presence of its distinct chemical functionalities. Similar peaks were previously reported in TCZ-loaded flexosomes (Ahmed et al. 2025g). The spectrum of oleylamine revealed prominent peaks at approximately 3333.0 cm⁻^1^, 2924.6 cm⁻^1^, and 2854.9 cm⁻^1^, corresponding to N–H and aliphatic C–H stretching vibrations. In contrast, the optimized formulation exhibited notable spectral shifts. New absorption bands emerged near 3333.0 cm⁻^1^ and 1710 cm⁻^1^, which align with Amide A (N–H stretching) and Amide I (C = O stretching) regions, respectively. These shifts suggest the formation of hydrogen bonding or electrostatic interactions, most likely between the amino functionalities of oleylamine and the carboxylic groups of oleic acid (Ahmed et al. 2024b). Moreover, the attenuation or disappearance of specific TCZ peaks within the spectrum of the optimized system implies successful drug encapsulation and a potential shift to a more dispersed molecular state within the vesicular structure. These spectral changes collectively confirm the integration of TCZ into the olaminosomal matrix and indicate structural stabilization through component interactions (Castro et al. 2016; Durukan et al. 2019). Nemr et al. reported similar findings, where the disappearance of the characteristic peaks of fenticonazole in FTIR analysis confirmed its successful entrapment within the positively charged Leciplex system (Nemr et al. 2025).

#### TEM microscopy

As depicted in Fig. 2, the TEM micrograph of the optimized formulation reveals well-defined, spherical vesicles with uniform size distribution and smooth surface morphology, indicative of structural integrity and successful formulation. The absence of aggregation in the micrograph indicates that the dispersion is physically stable and homogeneously distributed. Moreover, the vesicle dimensions observed through TEM closely matched those recorded via the Malvern system, confirming the formulation’s homogeneity and batch-to-batch consistency. The nanometric dimensions of the vesicles are considered advantageous for mucosal drug delivery, particularly within the vaginal environment, where small and flexible carriers can more efficiently interact with and permeate epithelial barriers. The presence of Span 60, a non-ionic surfactant, is presumed to enhance vesicle stability by lowering interfacial tension and promoting membrane fluidity, thereby supporting the structural integrity and adaptability of the vesicular system. Oleylamine, serving as a capping agent, stabilizes the vesicles and prevents overgrowth or aggregation, while oleic acid, a known penetration enhancer, improves membrane fluidity and drug diffusion. Limonene, a terpene with lipophilic characteristics, may disrupt lipid packing in the mucosal barrier, further facilitating drug transport (Ahmed et al. 2025d). Together, these components contribute to a formulation that is physically stable, nanosized, and well-suited for vaginal delivery, ensuring enhanced bioavailability and therapeutic performance of TCZ (Abd-Elsalam and ElKasabgy 2019; Asmaa Ashraf Nemr et al. 2024).

#### In vitro release and kinetic analysis

**Fig. 3:**
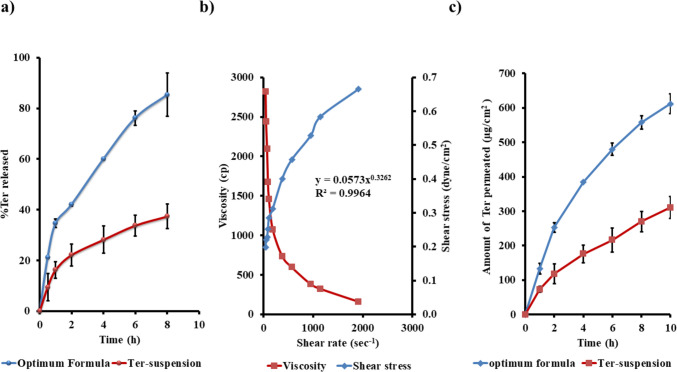
**a** In vitro release profile of the optimum formula compared to terconazole suspension. **b** Rheological characterization of the olaminogel. **c** Ex vivo permeation profile of olaminogel compared to plain terconazole gel

As illustrated in Fig. 3a, the release pattern of the optimized olaminosomal formulation exhibited a biphasic behavior. The initial phase was characterized by a rapid release, where approximately 40% of the drug was released within the first 2 h. This was followed by a second phase of gradual, sustained drug release over the remaining time period. This behavior is likely due to partial surface-associated drug contributing to the early release, while the remaining fraction is gradually released from the lipid bilayer matrix comprising oleylamine, oleic acid, Span 60, and limonene. These components collectively serve to restrict diffusion and enhance the solubility of terconazole. Unlike the TCZ suspension, the optimized formulation exhibited a more sustained and uniform drug release profile. Kinetic modeling revealed that the release pattern aligned most closely with the Higuchi model, indicating a diffusion-driven mechanism conducive to extended vaginal residence and localized therapeutic efficacy (El-Nabarawi et al. 2020; Higuchi 1961). Comparable results were previously reported by Fahmy et al., who observed a biphasic release profile for the hydrophobic curcumin (Abdurrahman M. Fahmy et al. 2025).

#### Mucoadhesion study

The mucoadhesive behavior of the optimized formula was investigated using mucin as a model to simulate the vaginal mucosal environment. The optimized formulation exhibited a zeta potential (ZP) of − 33.05 mV, primarily influenced by the presence of oleic acid, whose free carboxylic group significantly contributed to the negative surface charge (Abd-Elsalam and ElKasabgy 2019). In addition, oleylamine and the selected surfactants acted as capping agents, coating the nano-vesicles to prevent overgrowth and aggregation, thereby improving the colloidal stability of the system (Javed et al. 2020; Mbewana-Ntshanka et al. 2020). Upon mixing the optimized formulation with mucin, a notable shift in ZP values was observed, indicating strong electrostatic interactions between the nanoparticles and mucin chains. These interactions, supported by hydrogen bonding and van der Waals forces, suggest effective adhesion to the vaginal mucosa (Ahmed et al. 2025g). Such mucoadhesive capacity is expected to prolong residence time, reduce premature clearance, and enhance localized drug availability, collectively contributing to improved antifungal efficacy of the intravaginal nanocarrier (R. Albash et al. 2021; Takeuchi et al. 2005).

#### Stability study

The TCZ-loaded optimized formulation underwent short-term stability testing by being preserved at 4–8 °C over a 3-month period. During this period, no visible changes or signs of aggregation were observed, confirming the preservation of its physical appearance. As shown in Table 5, there were no statistically significant changes (*p* > 0.05) in the key physicochemical characteristics between the freshly prepared and stored formulations. Additionally, the calculated similarity factor (ƒ₂ = 64.70) confirmed a high degree of consistency between their in vitro release profiles, surpassing the commonly accepted threshold of 50 (Ahmed et al. 2025i). This strong alignment underscores the robust stability of the olaminosomal system. Oleic acid played a key role in generating a highly negative charge on the vesicle surface, and oleylamine, acting as a steric stabilizer, plays central roles in maintaining vesicle integrity. Furthermore, the small particle size enhances surface area exposure, facilitating improved colloidal stability. Together, these attributes validate the formulation’s suitability for vaginal delivery of TCZ, even after extended storage (Abd-Elsalam et al. 2018; Abd-Elsalam and ElKasabgy 2019).

**Table 5:** Effect of short-term stability on the optimum formula

Parameter	Fresh	Storage for 3 months at 4–8 °C	
		Value	Probability (***p***)*
**EE%**	82.11 ± 2.19	79.55 ± 0.94	0.268
**PS**	217.25 ± 6.58	209.60 ± 3.54	0.284
**ZP**	− 33.05 ± 0.92	− 35.45 ± 1.63	0.211

*EE%*, percent entrapment efficiency; *PDI*, poly-dispersity index; *PS*, particle size; *ZP*, zeta potential. ^*^One-way ANOVA analysis to compare between the freshly prepared and the stored optimum formula

#### Rheological studies

As illustrated in Fig. 3b, the optimized gel exhibited shear-dependent viscosity, with a notable decrease as shear rate increased consistent with pseudoplastic flow properties (Chang et al. 2002). This rheological behavior was confirmed by the power law model, which produced a flow behavior index (*n*) of 0.3262, signifying a shear-thinning (pseudoplastic) profile. Such non-Newtonian behavior is beneficial for vaginal drug delivery applications, as it allows the gel to reduce its viscosity under shear during application enhancing spreadability while recovering viscosity at rest, thus promoting retention at the site of administration (Chang et al. 2002; Yu et al. 2011). This increase in viscosity after application promotes mucoadhesion and prolongs residence time at the target site (das Neves et al. 2009). Additionally, the Carreau model showed excellent agreement with the experimental data (*R*^2^ = 0.9964), further confirming the non-Newtonian nature of the gel and their appropriateness for efficient vaginal administration with prolonged drug release (Yu et al. 2011). Younes et al. reported similar rheological behavior, where non-Newtonian pseudo-plastic flow contributed to improved retention and enhanced biological activity of the developed proniosomes (Nihal Farid Younes et al. 2024).

### Ex vivo permeation study

As illustrated in Fig. 3c, the ex vivo permeation results revealed that the optimized olaminogel markedly improved the transvaginal delivery of terconazole compared to the conventional gel. Over a 10-h period, the cumulative drug permeation (Q10h) achieved by the optimized formulation was approximately 612 ± 28.37 µg/cm^2^, indicating a significantly greater ability to facilitate drug passage through the vaginal mucosa, whereas the plain gel delivered only 310.59 ± 31.51 µg/cm^2^. In addition, the transmembrane flux (*J*ₘₐₓ) of the olaminogel was nearly twice that of the control (61.2 ± 2.83 vs. 31.1 ± 3.15 µg/cm^2^/h), yielding an enhancement ratio close to 1.97. These differences were statistically significant based on one-way ANOVA (*p* < 0.05), underscoring the optimized system’s superior ability to facilitate transvaginal drug transport.

The enhanced permeation is likely a result of the synergistic effects of the formulation’s key components. Oleylamine likely contributed to enhanced mucosal interaction through improved surface charge and reduced vesicle size. Oleic acid may have disrupted lipid packing within the mucosal layer, increasing membrane fluidity and permeability. Moreover, Span 60 might have facilitated drug transport by loosening tight junctions in the epithelial barrier. Altogether, these mechanisms highlight olaminosomes demonstrate strong potential as an effective nanocarrier system for localized and prolonged intravaginal delivery of terconazole (Sadek Ahmed et al. 2022).

### Microbiological evaluation

#### Minimum inhibitory concentration (MIC)

The antifungal effectiveness of the TCZ-loaded olaminosomal gel was evaluated using the broth microdilution method. The optimized formulation demonstrated a markedly enhanced antifungal effect, with a minimum inhibitory concentration of 3.9 µg/mL, significantly lower than that of the TCZ suspension, which exhibited an MIC of 125 µg/mL. This pronounced improvement highlights the role of the nanocarrier system in boosting drug efficacy. In contrast, the blank formulation (without active drug) exhibited no inhibitory effect, confirming that the formulation excipients lacked inherent antifungal activity.

### Minimum fungicidal concentration (MFC)

The fungicidal effect of both the TCZ suspension and the optimized olaminosomal formulation was assessed after 48 h of incubation at 28 ± 2 °C. While both samples showed fungicidal effects, the MFC value for the optimized formulation was significantly lower (125 µg/mL) compared to the plain drug suspension (500 µg/mL). This fourfold reduction in MFC further supports the superior antifungal efficacy imparted by the nanocarrier-based delivery system.

### Time to kill assay

To support the antifungal activity findings, the time-kill kinetics of both the pure drug and its optimized formulation against *Candida albicans* were further investigated. At their respective MICs, the optimized formulation (MIC = 3.9 µg/mL) achieved complete fungal eradication within 10 h of incubation, whereas the drug suspension (MIC = 125 µg/mL) required 30 h to achieve total killing of the fungal inoculum. This threefold reduction in the required time to kill the inoculated fungal inoculum underscored the optimized formula’s rapid action and, more importantly, highlights its enhanced antifungal potency compared to the parent drug. The substantial reduction in the fungicidal time aligns well with the earlier MIC and MFC findings, reinforcing the optimized formulation’s superior antifungal effect and potential enhanced therapeutic efficacy through accelerated fungus clearance.

### In vivo analysis

#### Histopathological evaluation

**Fig. 4:**
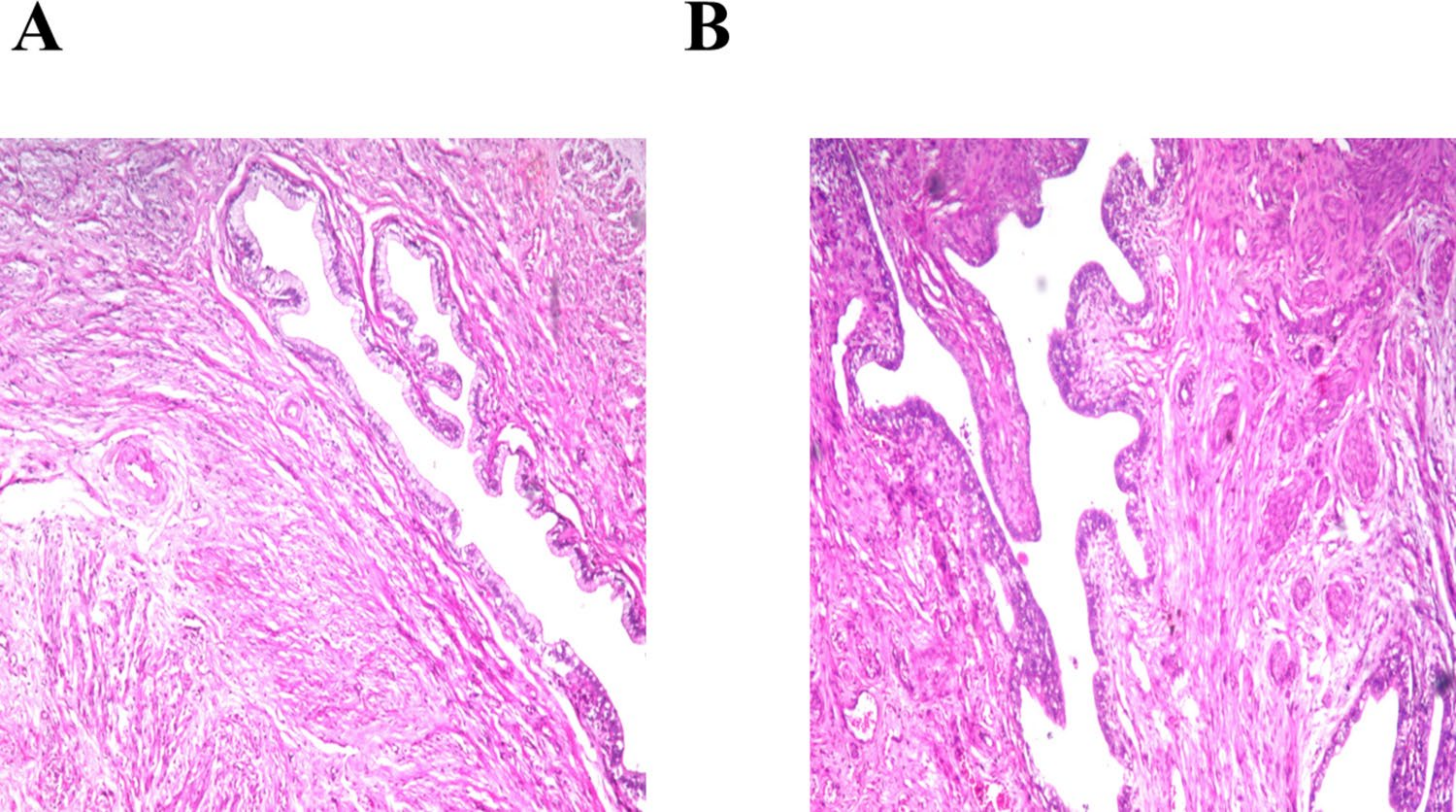
Histopathological sections of rabbits’ vaginal membrane after instillation of **A** normal saline solution (negative control) and **B** olaminogel

Microscopic examination of vaginal tissue, illustrated in Fig. 4, confirmed the biocompatibility and safety of the optimized olaminogel following intravaginal application. Tissues from the control group receiving normal saline (Fig. 4A) exhibited intact epithelial architecture, organized connective layers, and an absence of inflammatory response. Likewise, sections from rabbits treated with the optimized gel (Fig. 4B) revealed preserved epithelial integrity, with no observable signs of irritation, inflammation, or structural abnormalities, indicating that the formulation was well tolerated by the mucosal tissue. These results validate the non-irritant nature of the developed formulation and highlight its potential for safe intravaginal application. The absence of tissue damage is likely due to the strategic formulation of the vesicular system, particularly the inclusion of oleylamine and oleic acid, which enhance mucosal permeation while maintaining structural and functional integrity of the vaginal epithelium. These results support the potential of olaminosomes as a biocompatible and efficient vehicle for the targeted intravaginal delivery of TCZ (G. A. Abdelbary et al. 2017).

### In vivo permeation

**Fig. 5:**
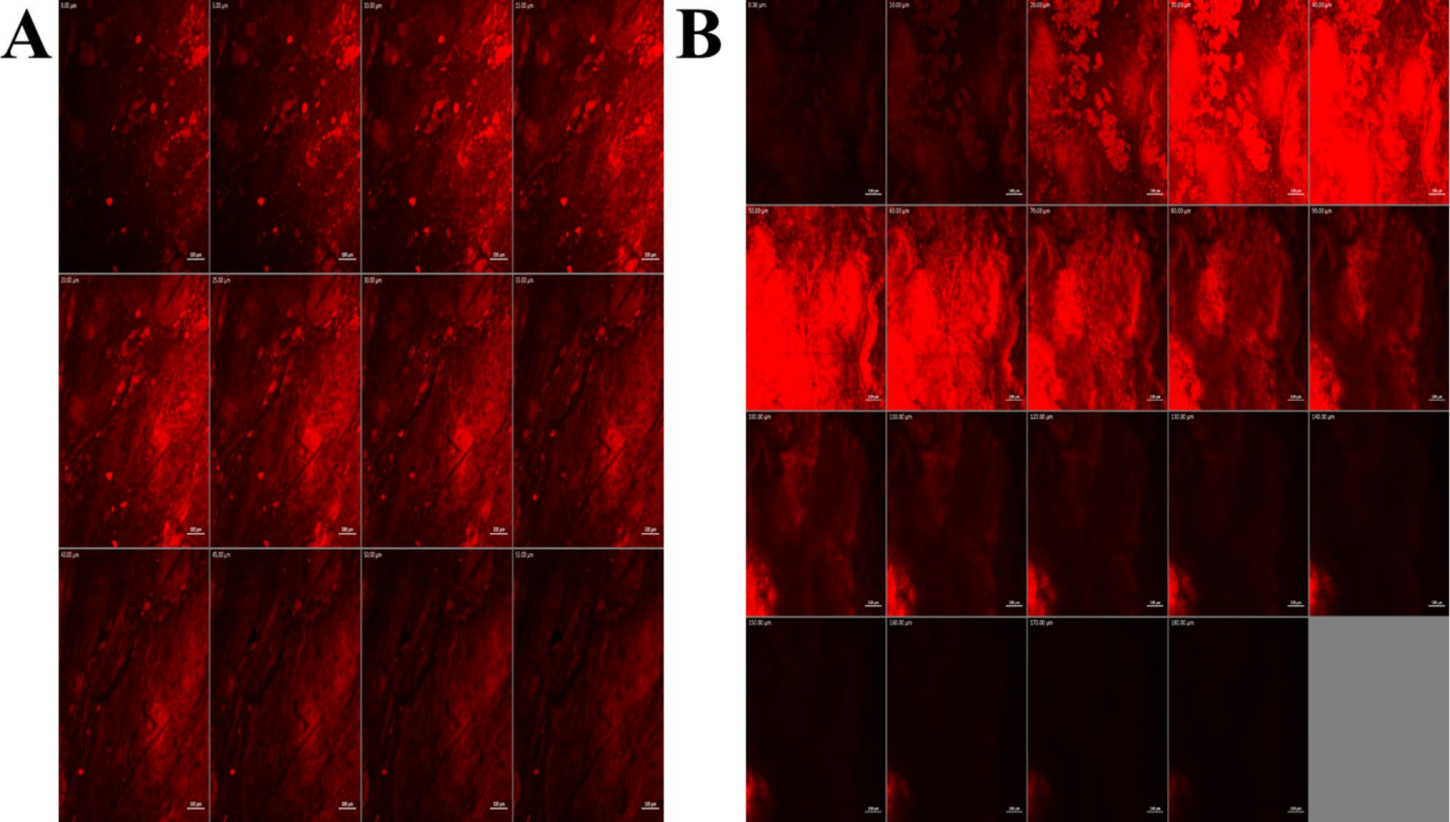
Confocal laser scanning micrographs of rabbits’ vaginal membrane after instillation of **A** RhB-loaded plain gel and **B** RhB-loaded olaminogel

An in vivo confocal laser scanning microscopy (CLSM) study was conducted to substantiate the improved mucosal permeation capacity of the optimized gel formulation. This analysis provided additional insight and supported the results obtained from earlier *ex-vivo* permeation studies. As shown in Fig. 5, the optimized olaminogel demonstrated markedly enhanced mucosal penetration, with fluorescence detectable up to a depth of around 180 µm. In contrast, the control gel displayed restricted diffusion, with fluorescence penetration limited to approximately 55 µm. The elevated fluorescence intensity and enhanced depth of distribution achieved by the optimized system indicate its superior ability to traverse mucosal barriers, further validating its potential for efficient local drug delivery.

## Limitations of the study

While this study highlights the potential of olaminogel as a novel nanocarrier for intravaginal delivery of terconazole, certain limitations should be recognized. First, the in vivo evaluation was limited to animal models, which may not fully mimic the physiological and pathological complexity of the human vaginal environment. Second, the long-term safety, pharmacokinetics, and possible mucosal alterations following repeated administration were not assessed and warrant further investigation. Third, although the formulation demonstrated superior antifungal efficacy with enhanced permeation and retention, the potential for resistance development and spectrum specificity against different fungal strains were not extensively explored.

## Conclusion

The ethanol injection method was employed to efficiently prepare olaminosomes in this research. The optimized formulation showed a nanoscale particle size (217.25 ± 6.58 nm), high entrapment efficiency (82.11 ± 2.19%), and a stable negative zeta potential (− 33.05 ± 0.92 mV), indicating strong colloidal stability. The vesicles exhibited sustained drug release in vitro, spherical morphology as observed by TEM, and excellent physicochemical stability throughout a 3-month storage period. FTIR spectroscopy confirmed the successful encapsulation of TCZ via observable shifts and attenuation in characteristic spectral peaks, indicating molecular interactions between TCZ and formulation excipients. Ex vivo permeation studies revealed significantly enhanced drug permeation through rabbit vaginal mucosa and flux compared to the plain gel. In vivo CLSM imaging further validated the superior mucosal penetration of the optimized formulation, achieving a penetration depth of 180 µm, markedly higher than the 55 µm observed with the control gel. Microbiological assays demonstrated enhanced antifungal efficacy, as evidenced by a substantial reduction in MIC and MFC values and a threefold faster kill time against *Candida albicans*. Histopathological evaluation demonstrated that olaminosomes are biocompatible and non-irritating, showing no signs of epithelial damage or inflammatory response. In summary, these findings affirm that olaminogel represents a robust and safe nanocarrier system capable of improving drug bioavailability, enhancing mucosal retention and penetration, and providing superior antifungal activity, making it a potential effective formulation for localized vaginal candidiasis management.

## Data Availability

All data generated or analyzed during this study are included in this published article.
